# Risk factors in hospital deaths in severely malnourished children in Kampala, Uganda

**DOI:** 10.1186/1471-2431-6-7

**Published:** 2006-03-16

**Authors:** Hanifa Bachou, James K Tumwine, Robert KN Mwadime, Thorkild Tylleskär

**Affiliations:** 1 Department of Paediatrics and Child Health, Makerere University, P O Box 7072, Kampala, Uganda; 2 FANTA/Regional Centre for Quality Health Care, Makerere University, Kampala, Uganda; 3 Centre for International Health, University of Bergen, Norway

## Abstract

**Background:**

Although the risk factors for increased fatality among severely malnourished children have been reported, recent information from Africa, during a period of HIV pandemic and constrained health services, remains sketchy. The aim of this study has been to establish the risk factors for excess deaths among hospitalized severely malnourished children of below five years of age.

**Method:**

In 2003, two hundred and twenty consecutively admitted, severely malnourished children were followed in the paediatric wards of Mulago, Uganda's national referral and teaching hospital. The children's baseline health conditions were established by physical examination, along with haematological, biochemical, microbiological and immunological indices.

**Results:**

Of the 220 children, 52 (24%) died, with over 70% of the deaths occurring in the first week of admission. There was no significant difference by sex or age group. The presence of oedema increased the adjusted odds-ratio, but did not reach significance (OR = 2.0; 95% CI = 0.8 – 4.7), similarly for a positive HIV status (OR = 2.6, 95% CI = 0.8 – 8.6). Twenty four out of 52 children who received blood transfusion died (OR = 5.0, 95% CI = 2 – 12); while, 26 out of 62 children who received intravenous infusion died (OR = 4.8, 95% CI = 2 – 12). The outcome of children who received blood or intravenous fluids was less favourable than of children who did not receive them. Adjustment for severity of disease did not change this.

**Conclusion:**

The main risk factors for excess hospital deaths among severely malnourished children in Mulago hospital include blood transfusion and intravenous infusion. An intervention to reduce deaths needs to focus on guideline compliance with respect to blood transfusions/infusions.

## Background

Under-nutrition is associated with >50% of all childhood mortality in developing countries [1,2], with the risk of mortality being 5–8 fold among severely compared to moderately malnourished children [3]. Because of the high risk of death, most severely malnourished children are managed in hospital. Unfortunately, the number of children hospitalised with severe malnutrition continues to rise in sub-Saharan Africa [4,5]. For instance, in Mulago Hospital, Uganda's national referral hospital, the number of children suffering from severe malnutrition increased from 11 to 45 per 1000 paediatric admissions between 1995 and 2002 [6]. Likewise, the case fatality rate increased from 15 to 24%. The case fatality rate in some African countries is >50% [7].

Factors contributing to the high case fatality in children hospitalized with severe malnutrition include acute bacterial infections, electrolyte imbalance, and micronutrient deficiencies [8-11]. Although prompt and appropriate treatment of severely malnourished children should reduce case fatality [9,12-14], no hospital study in sub-Saharan Africa has demonstrated a reduction of the case fatality to an acceptable international level of <5% – as reported, for example, in Asia [15], for which there can be several possible explanations. The difference observed may be in the stage at which the affected patients are brought for care, in the application of the clinical guidelines, or in the prevalence of HIV/AIDS [12,14,16,17].

Few studies have examined the potential risk factors for mortality among severely malnourished children admitted in African hospitals. This information is critical for the development of national guidelines for quality care of severely malnourished children. We therefore studied severely malnourished children admitted to Mulago hospital to map the mortality pattern, identify the risk factors for mortality and suggest potential interventions to reduce mortality. We also examined the difference in these factors among children HIV-infected severely malnourished children and those without HIV infection.

## Methods

### Study setting

Mulago Hospital, a national referral and teaching hospital, is situated in Kampala, the capital city of Uganda. The hospital provides various services ranging from primary to specialised care and serves urban, periurban and village populations from near and far districts. The Department of Paediatric and Child Health is one of the largest departments in the hospital, admitting over 10,000 children annually. The department has an Acute Care Unit which is open for admission 24 h a day. Admitted children are managed and observed for 24 h before being transferred to one of the four paediatric wards. Most of the children diagnosed with severe malnutrition are transferred to the Mwanamugimu Nutrition Unit either directly from the Acute Care Unit or the paediatric wards. This unit has two wards, a resuscitation ward with a bed capacity of 60 and a rehabilitation ward with a bed capacity of 25. The unit has been functional for nearly 4 decades. At the time of our study, the unit was supposed to use the Uganda national guidelines on management of severe malnutrition adapted from the WHO [18]. The national guideline includes optional measures in times of shortage or unavailability of supplies indicated in the WHO guidelines, such as: 1) high energy milk (100 Kcal/100 ml) made from fresh diary milk, vegetable oil and sugar, for use in place of F75 and F100; 2) standard WHO oral rehydration solution in the absence of ReSoMal; 3) use of multivitamin tablets in place of the combined mineral and vitamin mix. Copies of the national guidelines are usually made available to intern doctors and medical students.

### Study design and subject selection

**Figure 1:**
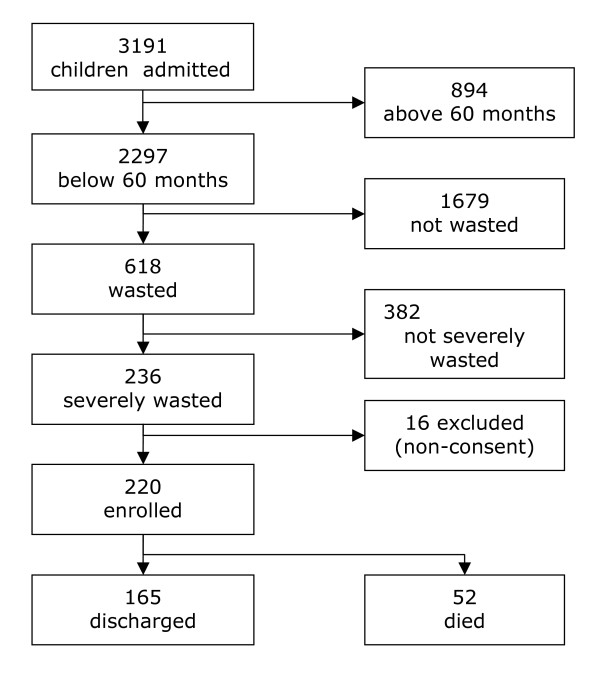
Study profile showing the process of enrolment of the 220 children with severe malnutrition below 60 months of age in Mulago hospital, Uganda, September – November 2003.

All children below the age of 60 months attending the department of Paediatrics and Child Health for acute illness between September 1 and November 15, 2003, were screened for severe malnutrition, using weight, height (or length for children less than 2 years) and presence of oedema. Measurements were taken in accordance with WHO standard techniques and compared with National Centre for Health Statistics (NCHS) reference population [19,20]. Severe malnutrition was defined as weight-for-height of <-3 z- score and/or presence of oedema (Figure 1). Children with length <49 cm were excluded from the study since the NCHS reference does not provide reference values for them. A total of 3,191 children were screened during the period. Of these, 236 (7.4%) were severely malnourished and their caregivers were informed of the study objectives and methods to be used, and requested for written consent to participate in the study. They were also counselled on having the HIV status of their children determined. Only children whose caregivers agreed to join the study by signing a consent form were enrolled.

A medical doctor trained in the study methodologies collected the children's demographic characteristics by interviewing the caregiver, filling in questionnaire, and determining their health characteristics using clinical history, physical examination and laboratory examinations of blood and urine specimens. A checklist was used to collect additional information from the patient's file immediately after the post-admission round by a team of doctors. Blood and urine specimens were drawn from each child whenever possible and the caregiver took the child for a chest x-ray examination.

The caregivers received pre-test and post-test counselling for HIV testing by a trained and experienced, mature multi-lingual counsellor. Caregivers of children who tested positive for HIV were counselled, and advised to attend the Paediatric Infectious Disease Clinic, a specialised outpatient clinic at the department for HIV positive children, for further management and subsequent follow-up. The clinic offers comprehensive HIV/AIDS care including free antiretroviral drugs. All other laboratory results were communicated to the caregivers and photocopies placed in the patients' files.

All enrolled children were followed-up daily, by an experienced study doctor on their respective wards who recorded the individual child's health development until outcome. Each child's daily management record was checked from files and recorded on observation forms, recording vital health signs, temperature, use of antibiotics, any fluids given and route of administration. Where necessary, the study provided any prescribed unavailable medication. Results of investigations were delivered to respective wards team promptly. Results needing urgent attention were communicated at once to the duty doctor.

### Laboratory investigations

#### Biochemical tests

An automated chemistry Express plus 550 analyzer (Hema-screen 18, LIHD 169, S/N 802723, Italy) was used to analyse serum protein, potassium and serum sodium were analysed using flame photometry with automated flame photometers IL 943.

#### Haematological tests

Haematological tests were done within 6 h of specimen collection. Haemoglobin measurement was based on the cyan-methaemoglobin method [21], and white cell counts was performed according to principle of impedance or electrical resistance. Results were obtained from print-out and expressed as cells per cubic millimeter (cell/mm^3^) and malaria parasites were examined from duplicate thick blood films from each specimen, using Field stain's, and examined under a microscope.

#### HIV Serology tests

Blood was taken in a 5 ml EDTA vacutainer tubes (Becton Dickinson, Franklin lakes, NJ USA) every morning between 8 – 11 am by venipuncture and transported within 4 h to Uganda Virus Research Institute (UVRI) laboratory, Entebbe, for serological testing. HIV testing was performed using standard HIV algorithm of two enzyme-linked immunoassays (EIA) in parallel. Western blotting, real-time polymerase chain reaction (RT-PCR) was performed to confirm a positive EIA test for children below the 18 months old and children with indeterminate results on EIA.

#### Blood and urine culture and sensitivity

Blood specimens for culture and sensitivity were cultured and sub-cultured on the blood agar, chocolate agar and crystal violet MacConkey agar plates and the plates incubated at 37°C for 24 h. The Kirby-Bauer diffusion method [22] was used to isolate, identify and characterize bacteria. Sensitivity to selected, commonly prescribed antibiotics were tested and graded as sensitive, intermediate or resistant.

Urine specimens with positive microscopic findings were cultured for bacterial sensitivity to commonly used antibiotics. Chest x-rays were taken to diagnose pneumonia, being reading and reported by a senior paediatric radiologist. The likely cause of death was determined by the research team based on clinical information, laboratory and x-ray findings. No Post-mortem examination was performed to verify the cause of death. We used clinical information, laboratory findings and x-ray reports to determine the likely cause of death.

### Ethical considerations

The study was approved by the institutional review boards in Norway (REK VEST), Makerere Medical School, Mulago Hospital and the Uganda National Council for Science and Technology (UNCST).

### Data Management

Raw data was cross-checked for completeness and correct labelling, and arranged in individual participants' fastened folders and stored securely in a filing cabinet. Data was entered and stored in Epidata . All data was stored securely in database accessible only to the research team. Stored data included patient identification inpatient or hospital number and study code. Anthropometric data was first analysed using the EPIINFO version 6 and later exported to SPSS and subsequent descriptive and statistical analyses.

### Statistical analysis

All statistical analyses were done using SPSS version 11.5. Univariate analysis by the Chi-squared test was used to measure association of each baseline characteristics with outcome, and logistic regression was used to explore the possibility of interactions. We conducted a multivariate analysis for all the variables using a hierarchical backward multiple regression to identify the variables most significantly associated with the outcome, and to adjust for the effects of age, sex and oedema (as dichotomous variables), as well as HIV status.

During modelling, variables were chosen according to their statistical significance of P <0.05, using odds ratio. Variables of clinical importance were included the initial model. Independent variables that persistently showed non-significant relationships with the dependent variable (death) during modelling were excluded from the final model. Age, HIV status and oedema were retained throughout modelling because of their primary interest in this study. We included interaction terms one at a time in the near final model and explored interactions within the multivariate model. We also conducted a survival function analysis for variables 'transfused', not 'transfused', as well as 'infused' and 'not infused'. Both unadjusted (Kaplan Meier) and adjusted (Cox) regressions were used.

## Results

During the study period 3191 children were admitted to the paediatric wards, figure 1. Of these, 220 severely malnourished children of <60 months were enrolled. Their characteristics are summarized in Table 1. Over 50% of the children were in the age group 6–24 months with median and interquartile range of 16 months (IQR 13.5 – 23.5) and about half the children had oedema, with or without a weight-for-height of <-3 SD.

**Table 1:** Characteristics of the 220 children below 60 months of age with severe malnutrition admitted to Mulago hospital, Uganda, September-November 2003

**Age group**	**Male**		**Female**		**Total**
			
Months	Oedema^a^	No oedema^b^	Oedema^a^	No oedema^b^	
**0 – 5.9**	0	2	1	3	6
**6 – 11.9**	14	13	7	9	43
**12 – 23.9**	38	37	20	26	121
**24 – 35.9**	8	7	5	6	26
**36 – 47.9**	5	2	3	3	13
**48 – 59.9**	3	2	4	2	11
**Total**	68	63	40	49	220

^a ^Bilateral pedal oedema with or without a weight-for-height of <-3SD (z-score)

^b ^Weight for height <-3 SD (z-score), but no bilateral pedal oedema.

The mean haemoglobin level (Hb) was 8 g/dL (SE 2.2) and the range 2 – 14.6. Over 80% of the 217 children with haemoglobin results were anaemic, 14 (6.5%) were severely anaemic (Hb< 5 g/dL) and 8 were very severely anaemic (Hb <4 g/dL; Table 2).

**Table 2:** Distribution of haemogobin levels of 217 children below 60 months of age with severe malnutrition admitted to Mulago hospital, Uganda, September-November 2003

**Haemoglobin levels (g/dL)**	**Number of children**	**Percentage (%)**
**< 4**	8	4
**4–4.9**	6	3
**5 – 8**	92	42
**8.1–10**	94	43
**>10**	17	8

**Total**	217	100

**Figure 2:**
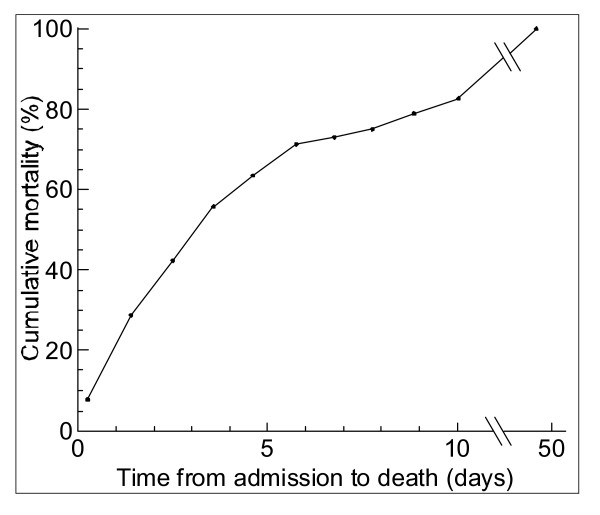
Cumulative mortality by time of death from admission of 52 deaths among 220 severely malnourished children in Mulago hospital, Uganda, September – November 2003.

Of the 220 children with severe malnutrition in hospital, 52 (24%) died, 107 (49%) were discharge against medical advice on achieving a target weight of 85% weight for height; and 59 (27%) self-discharged before target. The overall median duration from time of admission to time of death was 4 days (IQR 2 – 9), range 0 – 46 days. Fifteen children (29%) died in the first 48 h and 38 (73%) by the end of the first week (Figure 2).

**Figure 3:**
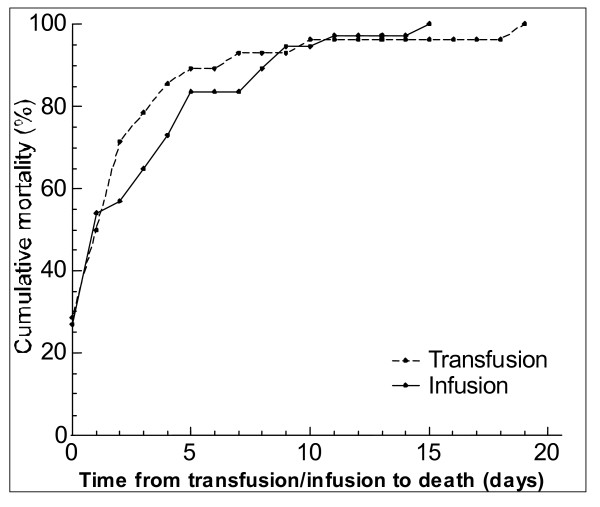
Cumulative mortality in the first week of admission of 28 and 37 children who were transfused and infused, respectively, by time from transfusion/infusion to death among severely malnourished children in Mulago hospital, Uganda, September – November 2003.

A total of 52 (23.6%) children received blood transfusion during management, of which 24 died. The median Hb level of the children who died was 8 g/dL (IQR 6.5 – 10), range 2.7 – 14.3. The median time from transfusion to death was one day (IQR 0 – 3), range 0 – 19 days. Twenty (71%) of these children died within the first 48 h after transfusion (Figure 3). Likewise, 62 (28%) children received intravenous fluid during management, of which 26 died. The median time from time of infusion to death was 1 day (IQR 0 – 11), range 0 – 15 days. Twenty one (56.8%) of the deaths occurred within the first 48 h from the time of infusion (Figure 3).

The main causes included septicaemia (10) and severe pneumonia (8). Others were tuberculous meningitis (3), drug reactions (2), severe anaemia (2), hypothermia/hypoglycaemia (2), hepatitis (1), cerebral malaria (1) and measles (1). For the rest of the children, fluid overload was the most likely cause. Of the 42 children who died in the first week, 17 (41%) tested positive for HIV-1 infection, 19 (45%) test negative HIV-1 infection and 6 (14%) had unknown HIV status. All the children were started on antibiotics at the time of admission. The majority were on a combination of ampicillin and gentamycin. Ten children were treated with ceftriaxone.

In the univariate analysis of the selected baseline and management characteristics, hypokalaemia, hypoalbuminaemia, transfusion intravenous fluid infusion were all associated with death (Table 3). There was no significant difference in mortality by sex, age-group (below 24 months or not), or by presence or absence of oedema.

**Table 3:** Univariate and multivariate analysis of factors associated with death

	**No of deaths/total (n = 220)**	**Unadjusted Odds ratio (95% CI)**	**Adjusted Odds ratio (95% CI)**
**Sex**			
Female	23/89	1	1
Male	29/131	0.8 (0.4 – 1.5)	1.0 (0.4 – 2.3)
**Age group**			
> 24 months	9/38	1	1
≤ 24 months	43/182	1.0 (0.4 – 2.3)	0.9 (0.3 – 2.4)
**Oedema**			
Absent	23/112	1	1
Present	29/108	1.4 (0.8 – 2.7)	2.0 (0.8 – 4.7)
**Axillary temperature < 35°C**			
No	44/169	1	
Yes	3/5	4.3 (0.7 – 26)	
**Diarrhoea**			
No	28/130	1	
Yes	24/90	1.4 (0.7 – 2.5)	
**Blood glucose < 3 mmol/L)**			
No	36/177	1	
Yes	7/18	2.5 (1.0 – 7.0)	
**Serum K^+ ^< 3.5 mmol/L**			
No	23/137	1	1
Yes	25/75	2.5 (1.0 – 4.8)*	2.4 (1.0 – 5.0)
**Serum Na^+ ^< 135 mmol/L**			
No	19/106	1	
Yes	29/106	1.7 (1.0 – 3.0)	
**Hb < 5 g/dL**			
No	46/203	1	
Yes	4/14	1.4 (0.4 – 4.6)	
**Malaria parasites in blood**			
No	45/186	1	
Yes	5/24	0.8 (0.3 – 2.3)	
**Bacteria isolate in blood**			
No	41/185		
Yes	10/32	1.6 (0.7 – 3.6)	
**Total serum protein < 6 g/dL**			
No	14/79	1	
Yes	34/133	1.6 (0.8 – 3.0)	
**Serum albumin < 3.5 mg/dL**			
No	3/42	1	1
Yes	45/170	4.7 (1.4 – 16)	4.3 (1.0 – 18)
**HIV Status**			
Negative	30/149	1	1
Positve	20/64	1.8 (0.9 – 3.5)	1.9 (0.8 – 4.4)
**Blood transfusion**			
No	28/142	1	1
Yes	24/52	3.5 (1.8 – 6.9)**	5.1 (2.2 – 12)***
**Intravenous fluid infusion**			
No	25/131	1	1
Yes	26/62	3.1 (1.6 – 6.0)*	4.8 (2.0 – 12)***

*p-value < 0.05; ***p-value < 0.001

The HIV positive children had a slight increase in the odds ratio for mortality but this was not significant (OR 1.8, 95% CI 0.93 – 3.5). None of the HIV infected children had been started on Antiretroviral (ARV) therapy during the study period. In the final multivariate model, we kept sex, age group, oedema and HIV status, all of which were still not significant. The 4 factors that were significant in the univariate analysis were serum potassium, serum albumin, transfusion and intravenous fluid infusion (Table 3). Blood transfusion and intravenous fluid infusion remained highly associated with death. We tested for all possible interactions in this final model and did not identify any that were significant.

**Figure 4:**
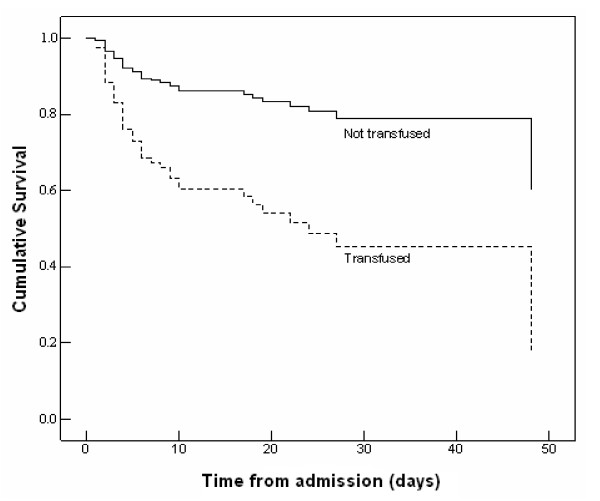
Cox regression survival curves of 194 severely malnourished children with complete data sets admitted to Mulago hospital, Uganda of which 52 died. The analysis was adjusted for the covariates retained in the final multivariate model (table 2) and stratified by transfusion status.

**Figure 5:**
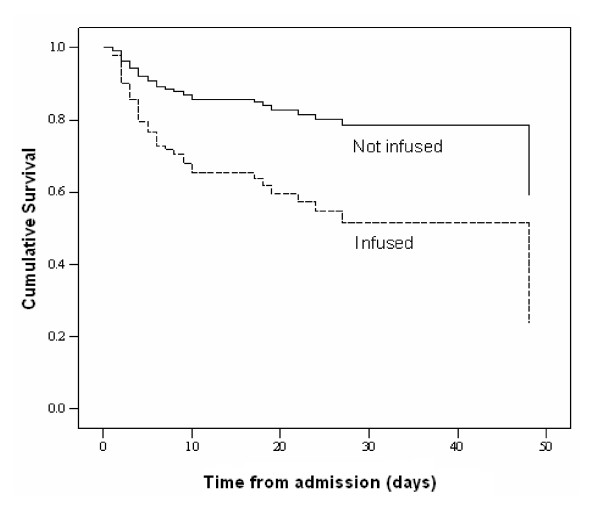
Cox regression survival curves of 194 severely malnourished children with complete data sets admitted to Mulago hospital, Uganda of which 52 died. The analysis was adjusted for the covariates retained in the final multivariate model (table 2) and stratified by infusion status.

We compared the risk of death in the two groups ('transfused' and 'not transfused') by survival analysis (Figure 4). The adjusted relative risk of the fatality for transfusion comparing 'transfused' and 'not transfused' was 3.3 (95%, CI 1.84 – 6.0). Similarly, the adjusted relative risk of the fatality for infusion comparing 'infused' and 'not infused' was 2.2 (95%, CI 1.36 – 4.78; see Figure 5).

In order to evaluate whether the children who were transfused/infused were more seriously ill and more in need of a transfusion and or intravenous fluid infusion, we analysed circumstances leading to their transfusion/infusion. According to most guidelines blood transfusion in severely malnourished children is recommended only if Hb is <4 g/dL (very severe anaemia) or they are in a state of shock. In Table 4, a cross tabulation of these children with or without severe anaemia (Hb<5 g/dL) by their transfusion status and outcome is presented. Of the 180 children with Hb ≥ 5 g/dL, 44 were transfused. Of these, 21 (48%) died, mostly within the first week of admission (80%). Among the 136 children who had Hb ≥ 5 g/dL and were not transfused, only 25 (18%) died. The odds ratio for death in the transfused group was 4.8 (95% CI 1.5 – 7.2) compared to the untransfused.

**Table 4:** Outcome of 191 severely malnourished children with known haemoglobin concentrations on admission to Mulago hospital, Uganda, September-November 2003; categorised by their transfusion status and indication for transfusion [25]

	**Transfusion may be indicated (<5 g/dL)**	**Transfusion not indicated (≥ 5 g/dL)**	**Total**
**Not transfused**			
Died	2 (50%)	25 (18%)	27
Alive	2 (50%)	111 (82%)	113
Total not transfused	4 (100%)	136 (100%)	140
**Transfused**			
Died	2 (29%)	21 (48%)	23
Alive	5 (71%)	23 (52%)	28
Total transfused	7 (100%)	44 (100%)	51

Table 5 shows a similar a cross tabulation of the children with or without severe dehydration (WHO definition) by their infusion status and outcome. Of the 183 children with no severe dehydration, about 30% were infused. Of these, 23 (43%) died, mostly within the first week of admission (75%). The odds ratio for death in the infusion group was 3.1 (95% CI 1.6 – 4.3) compared to the not infused group.

**Table 5:** Outcome of 193 severely malnourished children with diarrhoea at admission to Mulago hospital, Uganda, September-November 2003; categorised by their infusion status and indication for infusion [25]

	**Infusion may be indicated (Severe dehydration)**	**Infusion not indicated (No severe dehydration)**	**Total**
**Not infused**			
Died	0 (0%)	25 (19%)	25
Alive	1 (100%)	105 (81%)	106
Total not infused	1 (100%)	130 (100%)	131
**Infused**			
Died	3 (33%)	23 (43%)	26
Alive	6 (67%)	30 (57%)	36
Total infused	9 (100%)	53 (100%)	62

## Discussion

We have studied the risk factors for hospital death in 220 severely malnourished children below 60 months of age admitted to Mulago Hospital in Uganda, September – November 2003.

The main findings are that inappropriate use of transfusions and infusions seems to contribute significantly to case fatality. One third of the children were HIV positive but they did not have a statistically significant increased case fatality rate.

This study had several methodological advantages: 1) the use of reputable laboratories, especially for HIV detection at early age. 2) Combination of both clinical, laboratory and radiological assessments. 3) The consent to HIV testing was unexpectedly high (94%). 4) The analytical procedures used were adjusted for a number of possible confounders. There were, however, some drawbacks, such as the fact that children whose caregivers decided to leave hospital against medical advice were not followed up to determine their actual outcome. However, 50% of them left hospital after the first week of management. Another potential drawback is that all laboratory tests were performed once, on admission. Last, many caregivers did not take their children for x-ray examination.

Many of our results accord with previous findings in some African countries of high case fatality in hospitalized severely malnourished children [10-12,14,16,17,23]. Most deaths occurred in the first 7 days of admission. Irrespective of their HIV status, presence or absence of oedema, all severely malnourished children who received transfusion or intravenous fluid were at increased risk of dying compared to those who did not receive transfusion or intravenous infusion. Most of these deaths occurred soon after transfusion or/and infusion, within the first few days of admission. Therefore, fluid overload could be a plausible contributing factor to mortality. In addition, the survival experience of these children when transfused/infused was less favourable than of those who were not transfused/infused at all, as previously reported [7,12,15]. This corroborates the general advice to restrict transfusions or infusions of severely malnourished children (20, 22). Children who had severe anaemia or severe dehydration were few compared to the cases of transfusion and intravenous fluid infusions (Tables 4 and 5). In Bangladesh, restriction of intravenous fluids in management of severe malnutrition in children with diarrhea highly contributed to the reduction of case fatality to < 5% (15). In this study, severely dehydrated children were infused with low sodium high potassium fluids at 20 ml/kg body weight for the first 2 h, then at 10 ml/kg body weight for the next. The rehydration was changed to rice-based oral rehydration solution given at 10 ml/kg body weight per h for 2 h, after which 5 ml/kg body weight per h for the next 10 h. Therefore, transfusion and infusion should only be given to severely malnourished children when genuinely needed, e.g. in cases of haemolytic anaemia or shock and given cautiously with close monitoring of vital signs for heart failure.

Although both the WHO and the Uganda national guidelines for management of severe malnutrition recommend withholding transfusion unless a severely malnourished child's haemoglobin level is <4 g/dl [20], and withholding intravenous infusion unless a child has signs of severe dehydration or is in shock, our study shows that these guidelines were not followed. There are several possible reasons for non-compliance which may include; the practical difficulty in diagnosing severe dehydration in a severely malnourished child and the lack of adequate and fully functioning laboratories. Often, a doctor has to base her/his decisions to transfuse on the presence of severe pallor. Yet, pallor in state of severe malnutrition can be exaggerated. Distinguishing a case of AIDS from severe malnutrition per se on clinical grounds can be difficult and lead to inappropriate management.

No adequate association between HIV positive status and death was found, as reported in other studies in Africa [14,16,17]. The effect of HIV on mortality among severely malnourished children might have been overshadowed by the prominent effect of fluid overload. Appropriate management of severe malnutrition may unveil the effect of HIV on mortality.

High prevalence of bacteraemia, urinary tract infection and pneumonia found in this study have also been reported elsewhere [10,24,25]. However, the lack of association between infections and death could possibly be attributed to the fact that all severely malnourished children admitted to this hospital are routinely treated with intravenous broad spectrum antibiotics and that other factors were strongly associated with death. Details of the bacterial isolates and antibiotic sensitivity are presented and discussed elsewhere[26]

## Conclusion

The main risk factors for excess hospital deaths among severely malnourished children in Mulago hospital include blood transfusion and intravenous infusion. Any intervention to reduce deaths needs to focus on guideline compliance with respect to blood transfusions/infusions. This requires continued training of doctors and nurses in the paediatric wards in the management of severe malnutrition, especially where staff rotation (transfer) is common. Wide distribution and use of the WHO/national adapted version guidelines and user friendly job aids are advised.

## Competing interests

The author(s) declare that they have no competing interests.

## Authors' contributions

*A*ll authors participated in the design of the study, interpretation of the results, statistical analysis and writing the manuscript. HB supervised patient recruitment, follow-up and data collection. All authors read and approved the final manuscript.

## Financial support

Financial support was obtained from the NUFU supported project Essential Nutrition and child health, collaboration between the Department of Paediatrics and Child Health, Makerere University, Kampala, Uganda, and the Centre for International Health, University of Bergen Norway; NORAD and the Norwegian government Quota Program.

## Pre-publication history

The pre-publication history for this paper can be accessed here:


